# Synergistic Exposure of Rice Seeds to Different Doses of **γ**-Ray and Salinity Stress Resulted in Increased Antioxidant Enzyme Activities and Gene-Specific Modulation of TC-NER Pathway

**DOI:** 10.1155/2014/676934

**Published:** 2014-01-16

**Authors:** Anca Macovei, Bharti Garg, Shailendra Raikwar, Alma Balestrazzi, Daniela Carbonera, Armando Buttafava, Juan Francisco Jiménez Bremont, Sarvajeet Singh Gill, Narendra Tuteja

**Affiliations:** ^1^Plant Molecular Biology Group, International Center for Genetic Engineering and Biotechnology (ICGEB), New Delhi 110067, India; ^2^Department of Biology and Biotechnology “L. Spallanzani”, University of Pavia, 27100 Pavia, Italy; ^3^Plant Breeding, Genetics and Biotechnology Division, International Rice Research Institute (IRRI), 4031 Los Banos, Philippines; ^4^Department of Chemistry, University of Pavia, 27100 Pavia, Italy; ^5^Plant Biotechnology Lab, Division of Molecular Biology, Instituto Potosino de Investigación Científfica y Tecnológica (IPICYT), 78216 San Luis Potosí, SLP, Mexico; ^6^Stress Physiology and Molecular Biology Lab, Centre for Biotechnology, MD University, Rohtak 124001, India

## Abstract

Recent reports have underlined the potential of gamma (**γ**)-rays as tools for seed priming, a process used in seed industry to increase seed vigor and to enhance plant tolerance to biotic/abiotic stresses. However, the impact of **γ**-rays on key aspects of plant metabolism still needs to be carefully evaluated. In the present study, rice seeds were challenged with different doses of **γ**-rays and grown in absence/presence of NaCl to assess the impact of these treatments on the early stages of plant life. Enhanced germination efficiency associated with increase in radicle and hypocotyl length was observed, while at later stages no increase in plant tolerance to salinity stress was evident. APX, CAT, and GR were enhanced at transcriptional level and in terms of enzyme activity, indicating the activation of antioxidant defence. The profiles of DNA damage accumulation were obtained using SCGE and the implication of TC-NER pathway in DNA damage sensing and repair mechanisms is discussed. *OsXPB2*, *OsXPD*, *OsTFIIS,* and *OsTFIIS-like* genes showed differential modulation in seedlings and plantlets in response to **γ**-irradiation and salinity stress. Altogether, the synergistic exposure to **γ**-rays and NaCl resulted in enhanced oxidative stress and proper activation of antioxidant mechanisms, thus being compatible with plant survival.

## 1. Introduction

Rice is one of the leading food crops worldwide and increasing the rice production is expected to play a significant role in upgrading the economic status of developing countries in Asia and Africa. However, rice is highly sensitive to salt stress, especially at the early stages of plant development, and excess salt adversely affects all the plant's major metabolic activities causing yield losses [1]. Salt stress induces accumulation of reactive oxygen species (ROS), which result from altered metabolism in chloroplasts and mitochondria during stress, a condition which triggers increased levels of antioxidant enzyme activities [2]. Recent studies reported that plant exposure to different doses of gamma (*γ*)-irradiation may improve the tolerance to abiotic stress conditions, for example, salt and drought [3, 4].

Gamma radiation, composed of high energy photons, is a type of ionizing radiation, able to penetrate and interact with living tissues. It causes decreased growth rate and reproduction capacity along with DNA damage and morphological changes [5, 6]. However, irradiation with low doses is known to have stimulatory effects on plant growth, a concept referred to as hormesis [7].

Information concerning the use of *γ*-ray as a tool to improve seed vigour is still scanty. The process of seed priming carried out with osmotic agents induces the pregerminative metabolism, particularly the antioxidant response and DNA repair functions, leading to enhanced germination efficiency, a trait highly regarded for agricultural purposes [8]. Priming treatments might also improve stress tolerance in germinating seeds, leaving a sort of “stress memory” [9]. In contrast with its high applicability, little information is available on physical priming methods [10].

The exposure of biological systems to different types of ionizing radiation (IR), among which also *γ*-rays, sets in motion a number of steps, from the initial absorption of energy to the final biological injury. This can inflict damage to molecules by direct (e.g., ionization of key molecules like nucleic acids or proteins) or indirect actions (the energy is absorbed by the external medium, leading to ROS production) [11]. The major IR target is the water molecule. The primary reactions are excitation and ionization, which produce ionized water molecules (H_2_O^•+^) and free radicals (H^•^ and ^•^OH), also leading to chain reactions with production of secondary ROS (e.g., H_2_O_2_) [12]. On the other hand, increased ROS production can be induced through both natural and stress situations. These highly cytotoxic molecules can gravely disrupt the normal metabolism, causing oxidative damage to cellular components, but they are also known to act as critical signaling molecules in various processes [13]. For this reason, it is of utmost importance for plants, and all organisms in general, to maintain the cellular redox equilibrium. Organisms possess several antioxidant defence mechanisms which control the redox status of the cell, essential for normal physiological and biochemical functioning. Plant cells are equipped with several ROS detoxifying enzymes (peroxidases, catalase, superoxide dismutase, gluthatione reductase, etc) and other nonenzymatic antioxidants (ascorbate, glutathione, tocopherols, etc.) [14].

A critical point in the plant response to stress is related to the efficacy of DNA repair mechanisms. Among the different DNA repair processes, nucleotide excision repair (NER) represents a versatile pathway that acts on a wide range of substrates and it is divided into two subpathways: the Transcription Coupled-NER (TC-NER), which specifically removes lesions from the transcribed strands of active genes, and the Global Genome-NER (GG-NER), which deals with DNA damage throughout the remaining regions of the genome [15]. Genetic analysis of *Arabidopsis* mutants hypersensitive to UV light led to the identification of genes encoding some key components of the NER pathway, such as *AtXPB1*, *AtXPB2*,and *AtXPD*, which, similarly to their yeast and mammalian orthologs, function as helicases and are subunits of the transcription factor IIH (TFIIH) [16, 17]. XPB and XPD belong to the *Xeroderma pigmentosum* (XP) complementation group of proteins, which, if defective in humans, result in life-threatening hereditary diseases that are characterized by skin and eye photosensitivity manifested at different levels [18]. However, in plants, mutations in these genes are not lethal but they are characterized by developmental delay, lower seed viability, loss of germination synchrony and sensitivity to UV light [19, 20]. Recently, other transcription factors, namely, TFIIS (Transcription Elongation Factor II-S) and its homologue TFIIS-like, required during the transcription elongation phase for assisting RNAPII to escape from transcription pausing or arrests [21], were shown to be involved in the plant response to oxidative stress conditions as well as *γ*-ray exposure [22, 23]. Additionally, important functions of these genes were evidenced during seed dormancy as well as seed imbibition [24, 25].

In the present study, rice seeds, exposed to different doses of *γ*-rays delivered at both low and high dose rates, were grown in the absence/presence of a 100 mM NaCl solution. Doses and concentration were chosen to be compatible with plant survival. Seed imbibition and germination were tested and subsequently plantlets were analysed at 5 and 20 days after-germination. The level of oxidative stress was determined by measuring the lipid peroxidation, H_2_O_2_, proline and chlorophyll content, while the antioxidant capacity was tested through APX, CAT, and GR enzymatic activity and gene expression profiles. The occurrence of DNA damage was evidenced by using the comet assay and the expression profiles of *OsXPB2*, *OsXPD*, *OsTFIIS*, and *OsTFIIS-like* genes were evaluated. The impact of the synergistic exposure to *γ*-rays and salinity stress is discussed in relation to the antioxidant potential and DNA damage/repair processes.

## 2. Materials and Methods

### 2.1. Plant Material and Treatments

Rice (*Oryza sativa* var. *indica*) seeds, belonging to IR64 cultivar, were used in the present study. Dry seeds were exposed to different *γ*-ray total doses using a ^60^Cobalt source as follows: 25 and 50 Gy delivered at low dose rate (LDR; 0.28 Gy min^−1^); 100 and 200 Gy delivered at high dose rate (HDR; 5.15 Gy min^−1^). Subsequently, seeds were placed on Petri dishes supplied with filter paper for germination, in absence/presence of 100 mM NaCl. Water up-take was measured at 2, 4, 6, 8, 12, 24, and 36 h from the beginning of the experiment. For this, seeds were weighted prior to the imbibition as well as at the indicated time points and water uptake was calculated based on the weight differences. Radicle and hypocotyl length were measured 5 days after-germination. Plants grown in pots containing a mixture of vermiculite, sand, and peat moss in  1 : 1 : 1 ratio were kept under greenhouse conditions (30/20°C day/night temperature and 12 h photoperiod with 75–80% relative humidity). Also in this case, one lot of plants was watered, while another was grown in the presence of the 100 mM NaCl solution. The concentration of the NaCl solution was chosen to be compatible with plant life and development, based on previous studies carried out in our laboratory. Roots and leaves length were determined after 20 days of cultivation. The measurements were registered for 100 seedlings/plantlets for each treatment combination.

### 2.2. Lipid Peroxidation

The TBARS (Thiobarbituric Acid Reactive Substances) test is based on malondialdehyde (MDA) production during the oxidation of polyunsaturated fatty acids. The reaction between MDA and thiobarbituric acid (TBA) yields a reddish color, which corresponds to an absorbance maximum at 532 nm [26]. Plant material was homogenized extensively in liquid nitrogen with a mortar and pestle. The homogenized tissue powder (0.2 g) was suspended in 5 mL 0.1% TCA on ice and centrifuged at 10000 rpm for 10 min. Up to 1 mL supernatant, 2 mL 20% TCA, and 0.025 mL 0.5% TBA were added and the mixture was incubated at 95°C for 30 min, cooled on ice, and centrifuged at 10000 rpm for 10 min before reading absorbance at 532 nm subsequent to subtraction of nonspecific absorption at 600 nm. The MDA concentration was calculated using its extinction coefficient 156 mM^−1^ cm^−1^.

### 2.3. Histochemical Detection of Hydrogen Peroxide

The production of hydrogen peroxide (H_2_O_2_) in plant tissues was detected using 3′3-diaminobenzidine (DAB, Sigma-Aldrich) as described by Thordal-Christensen et al. [27]. Both 5-day-old seedlings and 20-day-old plants were used for the histochemical assay. As negative control plant tissues were treated with heat-shock (65°C for 20 min), the positive control is represented by the nontreated material. Plant material was incubated in 1% DAB solution for 1 h under vacuum and dark conditions. Subsequently, the material was further incubated overnight under agitation (200 rpm) and dark conditions. The plant material was washed with 75% ethanol for 3 times in order to remove the chlorophyll. Pictures were taken using a SONY NEX3E5 camera and the percentage of H_2_O_2_ accumulated in the plant tissue was calculated using the ImageJ software.

### 2.4. Proline Content

Proline was determined as described by Bates et al. [28]. Fresh tissues were homogenized in 10 mL of sulphosalicylic acid with pestle and mortar in ice cold bath. Homogenate was centrifuged at 10,000 g for 15 min. Two milliliters of filtrate was mixed with 2 mL of acid ninhydrin and 2 mL of glacial acetic acid. The mixture was incubated at 100°C for 1 h until the colored complex is developed in water bath and the reaction was terminated by cooling in ice. Subsequently, 4 mL of toluene was added to the coloured complex. Reaction mixture was vortexed for 15–20 s. Optical density of layer with chromophore was read at 520 nm. Proline content was estimated by using standard curve of L-proline.

### 2.5. Chlorophyll Content

For chlorophyll estimation, leaf samples (100 mg) were homogenized in 80% acetone. The homogenate was centrifuged (1,957 g) and the supernatant was collected. Absorbance was read at 662 nm (chlorophyll *a*), 645 nm (chlorophyll *b*), and 470 nm (xanthophyll and carotenoids) by using an Ultraspec 2100 Pro spectrophotometer (Amersham Biosciences). The pigment amount was calculated according to the subsequent formula [29]:

(1) Chl  a=  (11.75 A662)−(2.35 A645),Chl  b=(18.61 A645)−(3.960 A662),Chl  a+b=7.15  (A663)+18.71  (A646).

### 2.6. Enzymatic Activity

Samples were ground in liquid nitrogen prior to homogenization using ice cold enzyme specific buffer in pestle mortar. Homogenate was centrifuged at 15,000 g for 10 min at 4°C. Supernatant was separated and used for different enzyme assay. Protein content was determined by Bradford (1976) [30] using BSA as standard.

For the estimation of catalase (CAT; EC 1.11.1.6) activity, plant samples were homogenized in 50 mM phosphate buffer (pH 7.0) and 1 mM DTT (dithiothreitol). CAT activity was measured by using assay solution containing 50 mM phosphate buffer (pH 7.0), 33.5 mM H_2_O_2_, and 0.1 mL enzyme extract. Decrease in absorbance of H_2_O_2_ (*ε* = 39.4 mM^−1^cm^−1^) was recorded within 2 min at 240 nm [31]. One unit of CAT activity was defined as the amount of enzyme required to oxidize 1 *μ*mol of H_2_O_2_ per minute.

For the estimation of ascorbate peroxidase (APX; EC 1.11.1.11) activity separate extraction was carried out with the extraction buffer solution containing 100 mM phosphate buffer (pH 7.0), 0.1 mM EDTA, 1.0 mM ascorbate, and 1 mM DTT. APX activity was determined by monitoring the rate of hydrogen-peroxide-dependent oxidation of ascorbic acid in assay buffer that contained 50 mM phosphate buffer (pH 7.0), 0.5 mM ascorbate, and enzyme extract, in a total volume of 1 mL [32]. The rate of ascorbic acid oxidation was initiated by adding 10 *μ*L of 10% (v/v) H_2_O_2_ and the decrease in absorbance was monitored at 290 nm (*ε* = 2.8 mM^−1^cm^−1^) for 2 min. One unit of enzyme activity was defined as amount of enzyme required to oxidize 1 *μ*mol of ascorbate in one minute.

For glutathione reductase (GR; EC 1.6.4.2) estimation, tissues were homogenized in extraction buffer containing 100 mM phosphate buffer (pH 7.5) and 0.5 mM EDTA. The assay mixture contained 100 mM phosphate buffer (pH 7.5), 0.5 mM EDTA, 0.75 mM DTNB, 0.1 mM NADPH, and enzyme extract and reaction was initiated by adding 1 mM oxidized glutathione (GSSG) when 5,5-dithiobis (2-nitrobenzoic acid) (DTNB) was reduced by glutathione (GSH) to form TNB [33]. GR was assayed by monitoring the increase in absorbance at 412 nm (*ε* = 6.22 mM^−1^cm^−1^). One unit of enzyme was defined by amount of enzyme required to form 1 *μ*mol of GS-TNB min^−1^ by the reduction of DTNB.

### 2.7. Single Cell Gel Electrophoresis (SCGE)

Nuclei were extracted from treated material and untreated controls as described by Gichner et al. [34]. The suspension containing purified nuclei and a solution of 1% low melting point agarose in phosphate-buffered saline (PBS) at 37°C were mixed in equal volume. Two drops of the resulting suspension were then pipetted on agarose precoated slides and solidified on ice. Neutral SCGE, detecting the occurrence of double strand breaks (DSBs), was performed as follows: slides were incubated for 20 min at room temperature in high salt lysis buffer (2.5 M NaCl, 100 mM Tris-HCl pH 7.5, and 100 mM EDTA) to disrupt the nuclear membrane and subsequently electrophoresed for 8 min at 1 V cm^−1^ in TBE. Slides were subsequently washed in 0.4 M Tris-HCl (pH 7.5) three times for 5 min, rinsed in 70% ethanol (v/v) three times for 5 min at 4°C and dried overnight at room temperature. Next, slides were stained with 20 *μ*L DAPI (4′,6-diamidino-2-phenylindole, 1 *μ*g mL^−1^, Sigma-Aldrich). For each slide, one hundred nucleoids were scored, using a fluorescence microscope with an excitation filter of 340–380 nm and a barrier filter of 400 nm. Nucleoids were classified and results were expressed in % of DSBs according to Collins (2004) [35].

### 2.8. RNA Extraction and Quantitative Real-Time Polymerase Chain Reaction (QRT-PCR)

Total RNA was extracted from plant material by using TRIZOL reagent (Invitrogen) according to the manufacturer's instructions. A total quantity of 100 mg plant tissue was used. The RNA samples were treated with RNase-free DNaseI (Promega) to eliminate DNA contamination. The RNA quantification was performed using the PicoGene Spectrophotometer (Genetix Biotech). The absorbance ratios of the RNA samples at 260/280 nm and 260/230 nm were between 1.9 and 2.0. The quality of RNA samples was also verified on 1% agarose gel. Subsequently, cDNA was synthesized by using the iScript cDNA Synthesis Kit (BioRad), according to the manufacturer's instructions.

QRT-PCR reactions were performed in a 7500 Real Time PCR System apparatus (Applied Biosystems). Primers sequences ranging between 100 and 200 bp were designed by using the GeneScript Primer Design Program (https://www.genscript.com/ssl-bin/app/primer/) (Supplementary Table 1 in Supplementary Material available online at http://dx.doi.org/10.1155/2014/676934). For the cytosolic isoforms of *APX*, *CAT* and *GR* genes the most conserved regions were used in primer design. Endogenous gene was selected using GeNorm software [36]. The **α*-Tubulin *gene proved to be the most stable and was chosen as a reference [37, 38]. SSoFast EvaGreen Supermix (BioRad) was used for QRT-PCR reaction as indicated by the supplier. The amplification conditions were as follows: enzyme activation at 95°C for 30 s, denaturation step at 95°C for 10 s, and annealing/extension step at 57°C for 30 s and 60°C for 30 s, for 40 cycles. Fluorescence data was collected during the extension step and the specificity of qRT-PCR products was confirmed by performing a melting temperature analysis at temperatures ranging from 55°C to 95°C in intervals of 0.5°C. PCR fragments were run in a 2.5% agarose gel to confirm the existence of a unique band with the expected size. The Pfaff method [39] was used for data analysis.

### 2.9. Bioinformatic Analysis

The genomic sequences of *OsXPB2* (LOC_Os01g49680), *OsXPD* (LOC_Os05g05260), *OsTFIIS* (LOC_Os07g12630), and *OsTFIIS-like* (LOC_Os12g06850) genes were obtained from the Rice Genome Annotation Project funded by NSF (http://rice.plantbiology.msu.edu/). The protein domain search were performed in the NCBI Conserved Domain Database (NCBI-CDD; http://www.ncbi.nlm.nih.gov/Structure/cdd/cdd.shtml). The multiple sequence alignments was performed by using ClustalW Program (http://www.genome.jp/tools/clustalw/). The STRING computer service (http://string-db.org/) was used to determine the predicted protein-protein interaction.

### 2.10. Statistical Analysis

For each treatment combination, one hundred seeds were germinated and the analyses were performed in three independent replicas. Results were subjected to Analysis of Variance (ANOVA) and the means were compared by Student's *t*-test.

## 3. Results 

### 3.1. Effects of *γ*-Rays and Salinity Stress on Seed Water Uptake, Germination, and Plant Growth

**Figure 1:**
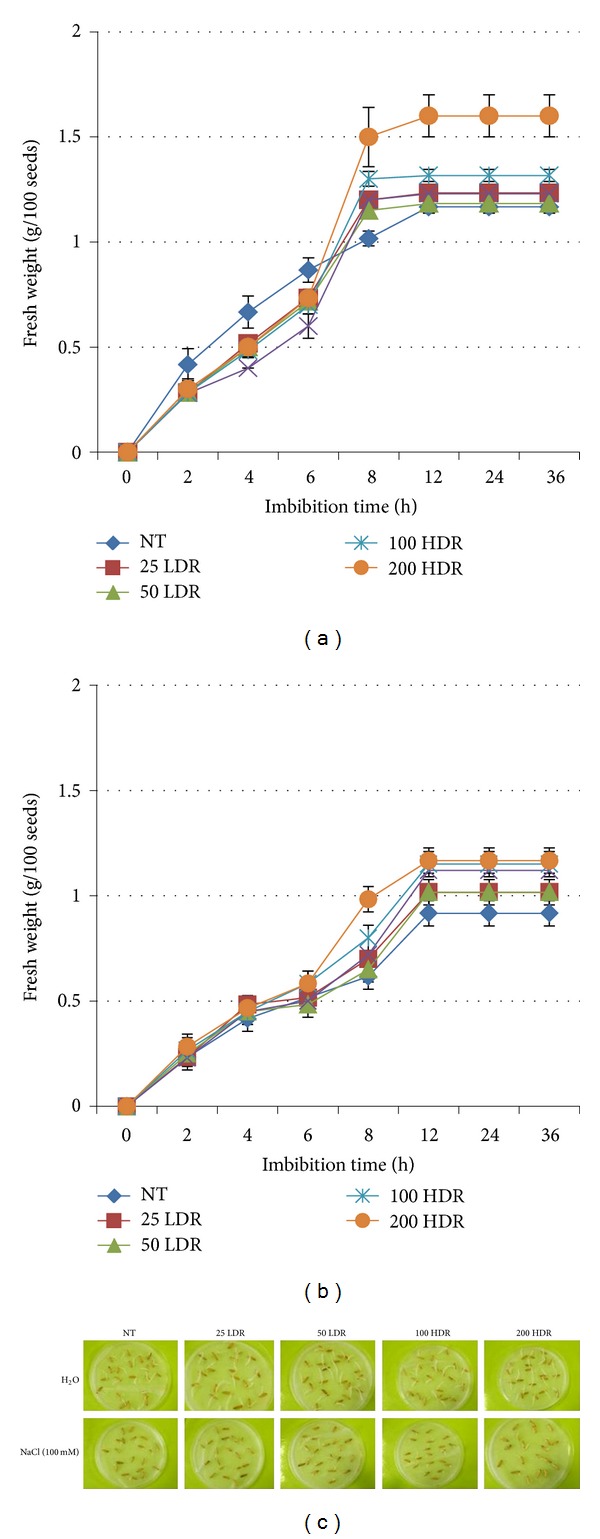
Water up-take measurements in irradiated rice seeds during imbibition carried out in presence of H_2_O (a) and 100 mM NaCl solution (b). For imbibition experiments, rice seeds were transferred to Petri dishes containing filter papers moistened with 2.5 mL distilled water and 100 mM NaCl solution and kept in a growth room at 30/20°C day/night temperature and 12 h photoperiod and a relative humidity (RH) of 75–80%. Fivedays after the beginning of the experiment, the germinated seeds were photographed (c) and germination efficiency was calculated. Values are expressed as mean ± SD of three independent replications with 100 seeds for each replication. NT: nontreated samples.

Rice seeds irradiated with 25 and 50 Gy delivered at a low dose rate, and 100 and 200 Gy delivered at a high dose rate, were rehydrated under physiological conditions and imbibed in a solution containing 100 mM NaCl. The gain in seed fresh weight occurring during the imbibition period and the germination efficiency were determined (Figure 1). In case of the seeds soaked in water, the phase I of imbibition (0–8 h) was characterized by a rapid increase in fresh weight (Figure 1(a)), and the Analysis of Variance showed significant (*P* < 0.001) differences among the sampling times. No significant differences in water up-take were observed when the LDR treatment was used, while the HDR doses induced a significant (*P* < 0.01) increase in the amount of absorbed water, especially at 200 Gy. On the other hand, a reduction in the gain in fresh weight was evidenced when imbibition occurred in the presence of NaCl (100 mM) (Figure 1(b)). The ANOVA showed a significant (*P* < 0.01) increase in fresh weight (from 0.23 ± 0.03 up to 0.70 ± 0.02 g per 10 seeds) which took place in the first 8 h after rehydration. In the irradiated seeds, the water up-take proved to be higher at all the tested doses as compared with the nonirradiated seeds. Two days after imbibition, seeds with protrusion of the primary root were considered germinated.  Figure 1(c) shows the germinated seeds in absence/presence of NaCl. The germination efficiency was measured in a previous study, where we observed an evident increase in germination efficiency in the seeds submitted to irradiation at all tested doses (from 80% in the control up to 98% at 200 HDR) [38]. By contrast, when seeds were imbibed in the NaCl solution, the germination efficiency was significantly lowered (45%), but an increase in germination efficiency was observed when seeds were irradiated [38].

Subsequently, the hypocotyl and radicle length were measured in 5-day-old seedlings imbibed both under physiological conditions and in the presence of NaCl solution (Supplementary Table 1). The stimulating effects of the irradiation treatments in seeds are still evident at this stage of plant growth. Significant increase (*P* < 0.01) in both radicle and hypocotyl length was visible and this enhancement was in a dose-dependent manner. As expected, the growth was slower when the seeds were imbibed in the NaCl solution. By contrast, when roots and leaves were measured after 20 days of cultivation in greenhouse, the stimulation effects of the irradiation treatments were no longer apparent (Supplementary Table 1). No significant differences were observed between the untreated samples and the IR treatments. The high levels of standard deviation might be due to the fact that these treatments normally induce high variability in the plant material. Additionally, the *γ*-ray treatments did not cause any apparent increase in the plant tolerance to salinity stress.

### 3.2. Exposure to *γ*-Rays and Salt Solution Causes Oxidative Stress in Rice Plantlets

**Figure 2:**
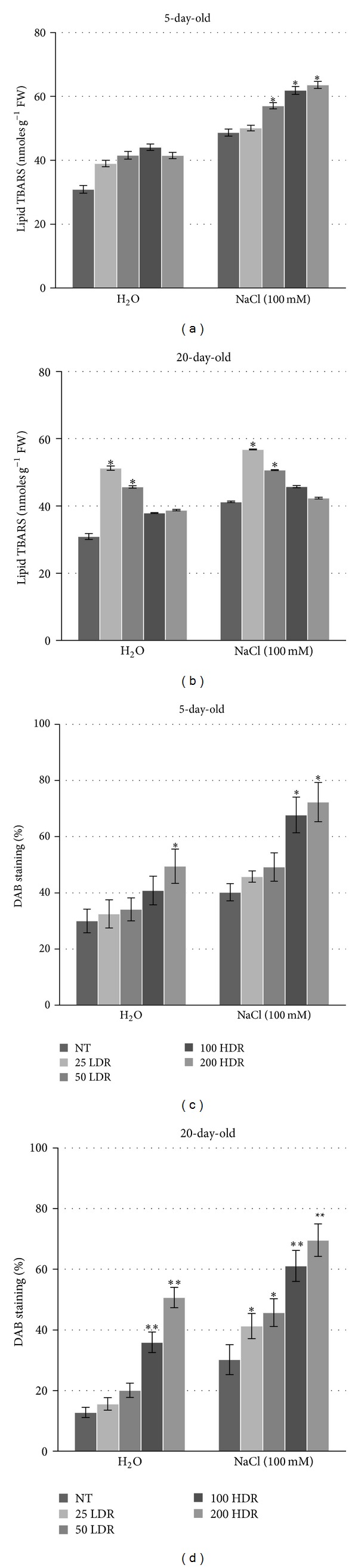
Lipid peroxidation measured by TBARS (thiobarbituric acid reaction) test in 5-day-old seedlings (a) and 20-day-old plantlets (b) grown from irradiated rice seeds in the absence/presence of NaCl. Hydrogen peroxide (H_2_O_2_) accumulation in 5-day-old seedlings (c) and 20-day-old plantlets (d) grown from irradiated rice seeds in the absence/presence of NaCl, as evidenced by DAB (3,3′-diaminobenzidine) staining. Values are expressed as mean ± SD of three independent replications with 100 seeds for each replication. Significant differences between control and treatments are represented by asterisk (*P < 0.05; **P < 0.01). NT: nontreated samples.

Lipid peroxidation (LP) and H_2_O_2_ levels are among the most representative tests to evaluate the oxidative damage in plant tissues. Under the present conditions, the lipid TBARS level was increased in the treated samples as compared with the untreated controls. In the 5-day-old seedlings, only a slight increase (around 5%) was observed when the irradiated seeds were germinated in the presence of water (Figure 2(a)). Significant (*P* > 0.01) increase in LP was evident when the seeds were grown in the presence of the 100 mM NaCl solution at all tested *γ*-ray doses, except the treatment with 25 LDR. As expected, the NaCl treatment induced higher lipid TBARS accumulation also in the control sample (nonirradiated). Conversely, when the 20-day-old plantlets were analyzed, the highest lipid TBARS level was registered with the 25 LDR treatment, both in the presence and absence of NaCl (Figure 2(b)).

H_2_O_2_ is a secondary ROS product that is normally present as a result of different metabolic activities, but it can also be formed during the ionization of biological tissues. In the present treatments, H_2_O_2_ accumulated in a dose-dependent manner both in the absence and presence of NaCl (Figures 2(c) and 2(d)). The highest levels were observed when the HDR treatments were used. Interestingly, higher levels of H_2_O_2_ were registered in the 5-day-old seedlings. This might be due to the fact that this compound is considered an important signaling molecule especially during seed germination and in the early seedling stages [39].

**Figure 3:**
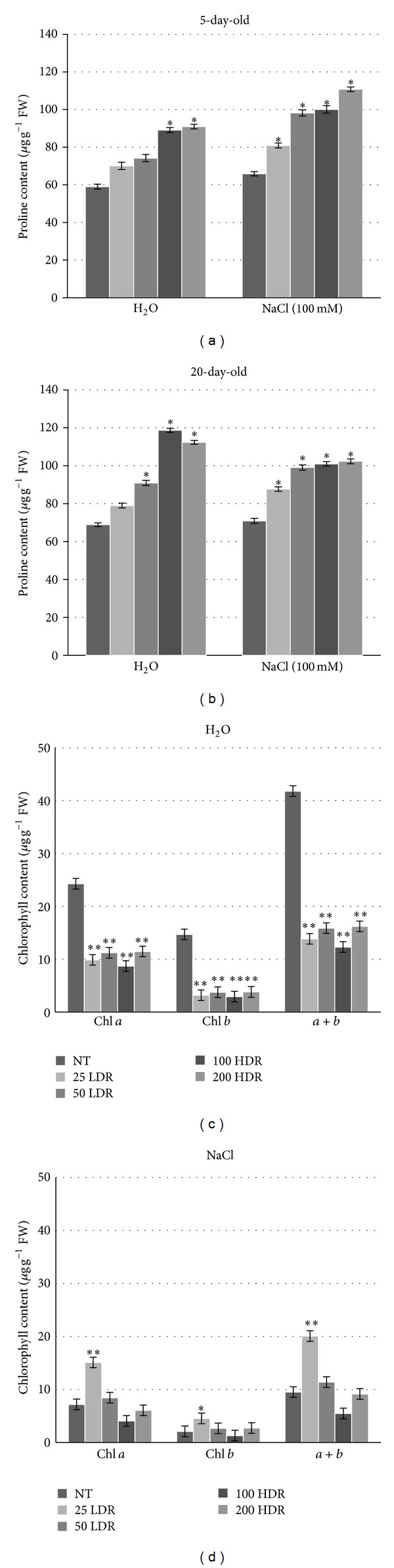
Proline levels in 5-day-old seedlings (a) and 20-day-old plantlets (b) grown from irradiated rice seeds in the absence/presence of NaCl. Chlorophyll content measurements in leaves of 20-day-old rice plantlets grown irradiated seeds germinated on water (c) and 100 mM NaCl solution (d). Both chlorophyll *a* and *b*, as well as total chlorophyll content (*a*/*b*), were measured. Values are expressed as mean ± SD of three independent replications with 100 seeds for each replication. Significant differences between control and treatments are represented by asterisk (*P < 0.05; **P < 0.01). NT: nontreated samples.

Additionally, the levels of proline, one of the essential amino acids considered to be an osmoprotectant, were also measured. Increased levels of proline were evidenced in rice plants grown from *γ*-irradiated seeds both in the absence and presence of the saline solution at all tested doses (Figures 3(a) and 3(b)). The highest proline accumulation was registered when HDR treatments were used.

Total chlorophyll content, as well as chlorophyll *a* and *b* was measured in the leaves of 20-day-old rice plantlets grown from irradiated seeds. When plantlets were cultivated only in the presence of water, a significant (*P* > 0.001) decrease in the chlorophyll content, was registered at all tested doses (Figure 3(c)). The reduction was more prominent in case of chlorophyll *b* content. On the other hand, when plantlets were grown in the presence of the NaCl solution, a significant 2.1-fold increase in chlorophyll *a* and *b*, as well as total chlorophyll content was evident when the 25 LDR treatment was used (Figure 3(d)). In case of the 50 LDR treatment, no differences were observed when compared to untreated control, while the HDR treatments resulted in a slight decrease in the chlorophyll levels.

### 3.3. Increased Antioxidant Enzymes Activity and Gene Expression Profiles in Response to *γ*-Rays and Salinity Stress

**Figure 4:**
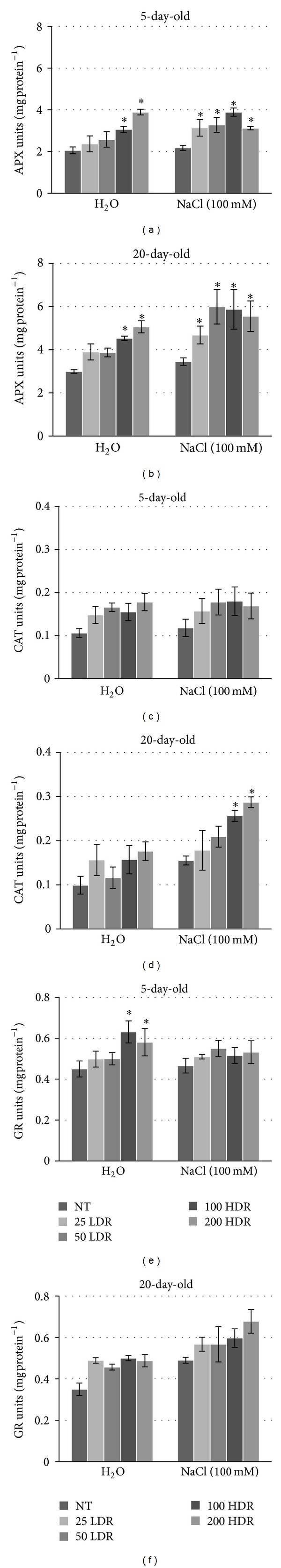
Effects of *γ*-irradiation on antioxidant enzymes activity in 5-day-old seedlings and 20-day-old plantlets grown in the absence/presence of NaCl; (a, b) APX activity; (c, d) CAT activity; (e, f) GR activity. Values are expressed as mean ± SD of three independent replications with 100 seeds for each replication. Significant differences between control and treatments are represented by asterisk (*P < 0.05). NT: nontreated samples.

Ascorbate peroxide (APX), catalase (CAT), and glutathione reductase (GR) enzymatic activities were evaluated in rice seedlings and plantlets grown from *γ*-irradiated seeds in the absence/presence of NaCl (Figure 4). Among the three antioxidant enzymes, APX showed the highest enzymatic activity. In irradiated seedlings germinated on water, a progressive increase was observed only with the HDR treatment with the highest peak (1.9-fold) at 200 HDR, while in the presence of NaCl enhanced activity was observed also with the LDR treatments (Figure 4(a)). When the 20-day-old plantlets were analyzed, the APX enzymatic activity was further enhanced, especially in the presence of NaCl (Figure 4(b)). However, in this case, increased activity was also evidenced in the non-treated controls as compared with the seedling stage. As for the CAT activity in seedlings, it ranged from 0.1 ± 0.01 mg protein^−1^ in nontreated samples (NT) up to 0.17 ± 0.02 mg protein^−1^ in response to irradiation and NaCl treatments (Figure 4(c)). In 20-days-old plantlets, the CAT activity was exponentially increased in a dose-dependent manner in the presence of NaCl (Figure 4(d)). As for the GR enzymatic activity, in seedlings no significant differences were observed between the control and treatments, except for HDR treated seeds imbibed in the presence of water where an approximately. 1.4-fold enhancement was evident (Figure 4(e)). In addition, the GR enzymatic activity was not significantly increased in the 20-day-old plantlets, even if a slight increment was observed (approximately 0.7-fold 200 HDR NaCl) (Figure 4(f)).

**Figure 5:**
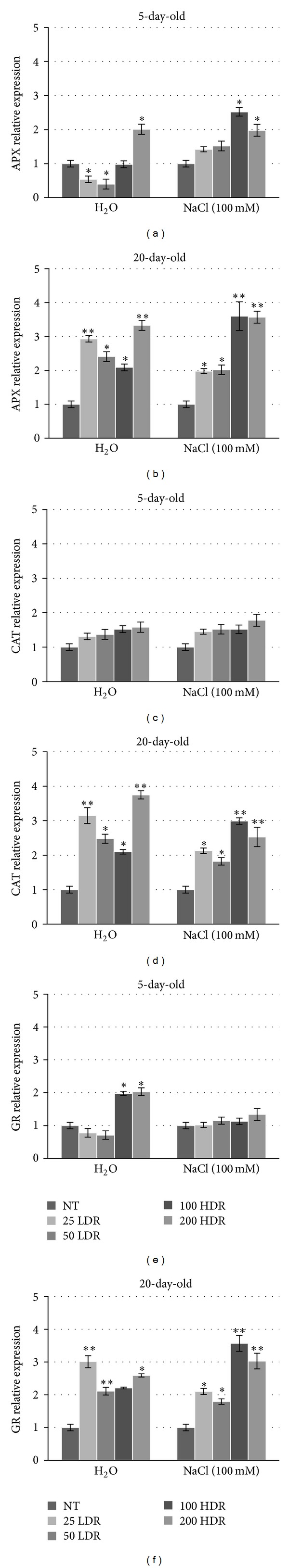
Gene expression profiles of the antioxidant genes in 5-day-old seedlings and 20-day-old plantlets grown in the absence/presence of NaCl; (a, b) cytosolic APX activity; (c, d) CAT activity; (e, f) GR activity. Values are expressed as mean ± SD of three independent replications with 100 seeds for each replication. Significant differences between control and treatments are represented by asterisk (*P < 0.05; **P < 0.01). NT: nontreated samples.

In order to test if the enzymatic activity correlates with the gene expression profiles, the cytosolic isoforms of APX, CAT, and GR gene expression patterns were evaluated by means of QRT-PCR (Figure 5). In 5-day-old seedlings germinated on water, the APX gene was downregulated up to 2.5-fold in response to LDR irradiation, while it presented a slight upregulation (1.0-fold) under 200 HDR treatment. When the irradiated seeds were germinated in the presence of NaCl, APX transcript was significantly increased (approximately 1.5-fold) only in response to HDR treatments (Figure 5(a)). However, when the APX transcript level was evaluated in 20-days-old plantlets, a significant increase was evident with all irradiation doses and both in absence and presence of NaCl. The highest increase (up to 2.6-fold) was observed with the HDR treatments in the presence of NaCl (Figure 5(b)). As for the CAT expression profiles in seedlings no significant differences between control and treatments were evidenced (Figure 5(c)), while significant up-regulation (up to a maximum of 2.8-fold in 200 HDR H_2_O) was observed in case of the 20-day-old plantlets (Figure 5(d)). Finally, the GR mRNA levels were slightly increased (1.0-fold) only in seedlings germinated on water from HDR-treated seeds (Figure 5(e)). On the other hand, also in this case, the gene was significantly up-regulated throughout all the tested conditions in 20-days-old plantlets. The highest GR levels (up to 2.5-fold) were registered in HDR-treated seeds and NaCl exposure (Figure 5(f)).

Taken together, the present results show that the increase in antioxidant enzymes activities well correlates with the up-regulation of APX, CAT, and GR genes, especially when the HDR-treatments and the 20-day-old plantlets are concerned.

### 3.4. DNA Damage Profiles during the Synergistic Exposure to *γ*-Rays and Salinity Stress

**Figure 6:**
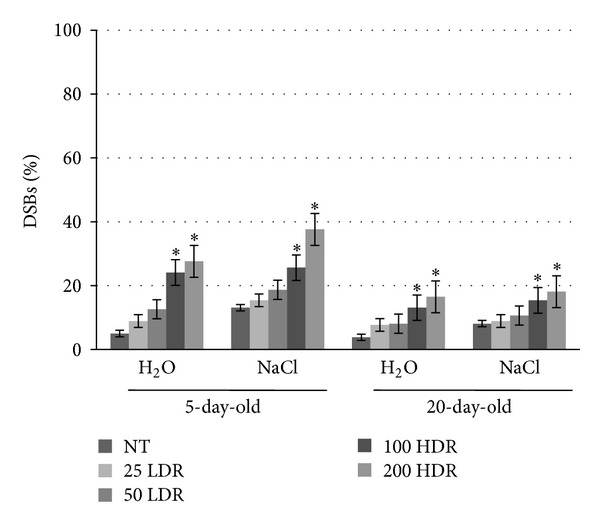
Percentage of double strand breaks (DSBs%) as measured by the neutral version of SCGE (Single Cell Gel Electrophoresis) assay in 5-day-old seedlings and 20-day-old plantlets grown from *γ*-irradiated rice seeds in the absence/presence of 100 mM NaCl. Values are expressed as mean ± SD of three independent replications with 100 seeds for each replication. Significant differences between control and treatments are represented by asterisk (*P < 0.05). NT: nontreated samples.

The percentage of DSBs was calculated based on the results obtained by using the neutral version of SCGE assay (Figure 6). The basal percentage of DSBs in non-treated control was 5% in 5-day-old seedlings and 3.8% in 20-day-old plantlets. When the irradiated seeds were germinated in the presence of water, a dose-dependent increase in the DSBs level was evidenced in seedlings, with significant differences for the HDR treatments (24.1% and 26.7%). The same pattern was also observed when the seeds were germinated in the presence of NaCl (25.6% and 37.6%), underling the fact that in this case the percentage of DSBs was higher than when the irradiated seeds were germinated only on water. It is interesting to note the fact that the 20-day-old plantlets presented a decrease in DSBs accumulation as compared with the 5-day-old seedlings. In the absence/presence of NaCl the percentage of DSBs at 200 HDR was 16.5% and 18.1%, respectively. These results underline that fact that DNA damage repair is activated in this timeframe. It is also important to note that the DNA damage levels observed under the present conditions are still compatible with plant's life.

### 3.5. Genes Involved in TC-NER Pathway are Differentially Modulated in Response to *γ*-Rays and Salinity Stress

In order to acquire more information on the OsXPB2, OsXPD, OsTFIIS, and OsTFIIS-*like* functions an *in silico* analysis was performed using bioinformatic programs. The gene and protein sequences were obtained from the Rice Genome Annotation database, by blasting the known sequences from *A. thaliana* and *M. truncatula*. Results from the bioinformatic analysis are provided and discussed in the Supplementary Material (Supplementary Tables 3 and 4 and Supplementary Figures  1 and  2).

**Figure 7:**
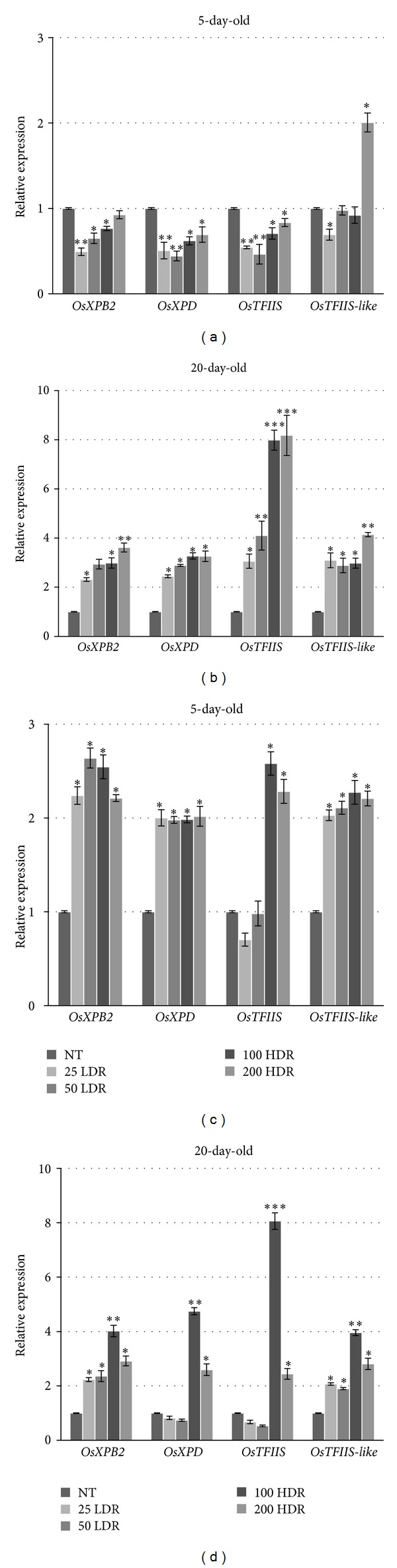
Expression profiles of *OsXPB2*, *OsXPD*, *OsTFIIS*, and *OsTFIIS-like* genes in response to LDR and HDR *γ*-ray exposure in rice seeds were estimated by QRTPCR; 5-day-old seedlings germinated in the absence/presence of NaCl (a, c); 20-day-old plants grown in the absence/presence of NaCl (b, d). Quantification of each gene, expressed as relative mRNA level compared with a control, was calculated after normalization to **α*-Tubulin *gene. For each treatment, data (±SD) were derived from three independent replications. Significant differences between control and treatments are represented by asterisk (*P < 0.05; **P < 0.01; ***P < 0.001). NT: nontreated control.

The expression patterns of *OsXPB2*, *OsXPD*, *OsTFIIS* and *OsTFIIS-like* genes were evaluated by QRT-PCR in rice 5-day-old seedlings and 20-day-old plantlets grown from irradiated seeds. When irradiated seeds were germinated in the presence of water, the *OsXPB2* gene was significantly down-regulated in response to 25 LDR, 50 LDR, and 100 HDR (2.0-, 1.5-, and 3.1-fold, resp.), while no significant changes were observed when the highest dose was used (Figure 7(a)). A similar trend was observed also in case of the *OsXPD* gene. However, the gene was slightly down-regulated also in response to the highest dose (1.4-fold, 200 HDR). The *OsTFIIS* gene expression was more affected by the irradiation treatments, being significantly down-regulated (1.8- and 2.2-fold) in response to 25 and 50 LDR, as well as to 100 and 200 HDR treatments (3.5- and 1.2-fold, resp.). As for the *OsTFIIS-like* gene, its expression pattern was more intriguing. At the seedling stage, the gene transcript decreased with 1.9-fold in response to 50 HDR treatment and increased with 1.0-fold when 200 HDR dose was used (Figure 7(a)). Conversely, when the gene expression patterns were registered in the 20-day-old plantlets, all the genes were significantly up-regulated (Figure 7(b)). The highest transcript accumulation (7.2-fold) was observed for the OsTFIIS gene with the higher doses. Overall, the QRT-PCR analysis revealed a trend of down-regulation of the TC-NER genes at the seedling stage, followed by dose-dependent up-regulation in the 20-day-old plantlets.

When the *OsXPB2*, *OsXPD*, *OsTFIIS*, and *OsTFIIS-like* gene expression patterns were tested in response to both irradiation and salinity stress (100 mM NaCl) the general outlook changed, since the genes were mostly up-regulated in seedlings as well as in plantlets (Figures 7(c) and 7(d)). The *OsXPB2 *mRNA levels were enhanced in seedlings, with approximately 2.0-fold at all tested doses. In the 20-day-old plantlets, the highest transcript levels were registered when the 50 and 100 HDR doses were used (2.7- and 3.0-fold, resp.). In the 5-day-old seedlings, the *OsXPD* gene presented the same trend as *OsXPB2*. However, when the plantlets were analysed a significant up-regulation was observed only in response to the highest doses (100 HDR, 3.7-fold; 200 HDR, 1.6-fold). The *OsTFIIS* mRNA levels were increased in seedlings only in response to HDR treatment (100 Gy, 2.6-fold; 200 Gy, 2.3-fold). By contrast, in the 20-day-old plantlets, its expression showed downregulation when the LDR treatment was used (1.5- and 1.8-fold). However, the use of the highest doses resulted in significant up-regulation, especially at 100 HDR (7.1-fold). The *OsTFIIS-like* gene presented same pattern of expression as *OsXPB2* and *OsXPD* genes in seedlings. When the plantlets were examined, the up-regulation was maintained and peaked at 100 HDR with a 3.0-fold increased.

Altogether, the concomitant use of *γ*-rays and osmotic agent resulted in differential expression of the genes involved in TC-NER pathway. In this case, the dose-dependent pattern observed when only irradiation was applied was no longer apparent.

## 4. Discussion

In the present study, the effects of LDR and HDR *γ*-rays were investigated in relation to water up-take and seed germination efficiency, salinity stress tolerance, and antioxidant capacity, as well as dynamics of DNA damage and repair competences. Physiological symptoms have been described by several researchers in a large number of plants exposed to *γ*-rays [6, 40]. The symptoms frequently observed in the low or high dose irradiated plants were enhancement or inhibition of germination and seedling growth, differential antioxidant responses, and DNA damage accumulation [4, 6, 40].

When rice seeds were treated with *γ*-rays, water up-take was always enhanced during imbibition carried out both under physiological conditions and in the presence of NaCl as osmotic agent. Usually, at the beginning of imbibition, massive leakage of cellular solutes, caused by rapid rehydration, accelerates germination by lowering the concentration of inhibitors. Following water up-take and increased seed size, the seed enters a phase during which no changes in the water content occur (plateau phase), though the water up-take continues until radical protrusion is observed. When seed imbibition takes place in the presence of an osmotic agent, the rehydration rate is decelerated and this event reduces the level of cellular damage that normally occurs as a consequence of the rapid water entry into dry seeds [8, 25]. The augmentation in water up-take resulted in enhanced seed germination and an increase in radicle and hypocotyl length with all tested doses. Contrasting reports have been provided, concerning the effects of **γ**-rays on seed germination efficiency. Borzouei et al. [41] reported that wheat germination efficiency was inhibited at doses in the 100–400 Gy range, while Singh and Datta [42] showed that lower doses (10–100 Gy) improved the germination potential of wheat seeds. Similarly, exposure to 1 or 2 Gy slightly stimulated the growth of *Arabidopsis* seedlings while an inhibitory effect on growth was observed using 50 Gy [6]. When considering the potential use of *γ*-rays for seed priming strategies, the field of research is quite limited. However, other biophysical methods such as laser beam or magnetic and electromagnetic stimulation were proven to be effective [10]. Surprisingly, the stimulatory effects of *γ*-ray treatments observed in rice seedlings did not persist in the older plantlets. When the length of roots and leaves was measured in rice plantlets after 20 days of cultivation, no differences were observed between the untreated control and the plants grown from irradiated seeds, both under physiological conditions and in the presence of the osmotic agent. This finding is in contrast with the reports describing alleviation of salinity stress upon preexposure to *γ*-rays published on rice and cowpea [4, 43].

Subsequently, we have showed that *γ*-ray exposure as well as the synergistic action of **γ**-rays and salinity stress resulted in oxidative stress in both 5- and 20-day-old rice plantlets. This was proved by the increase in LP and H_2_O_2_ accumulation, especially when the rice seeds were irradiated with HDRs and in the presence of NaCl. Additionally, a significant decrease in chlorophyll content was evidenced in rice plants in response to all treatments, except for the 25 Gy LDR condition used in concomitance with the osmotic agent. Modification in pigment structure and content were previously reported in plants treated with *γ*-rays, such as in lettuce (*Lactuca sativa*) where seed exposure to doses ranging from 2–30 Gy enhanced the level of photosynthetic pigments, while at higher doses (up to 70 Gy), a decrease was noticed [44]. Considering the present results, it is also important to underline the observed differences between the LDR and HDR treatments, where, generally, under LDR the induced stress was reduced. Nevertheless, under the tested conditions, the rice plantlets showed proper activation of the antioxidant defence, as demonstrated by the increase in APX, CAT, and GR enzymatic activities which well correlated with up-regulation of the corresponding genes. Usually, H_2_O_2_ is scavenged by CAT as well as different classes of peroxidases, while APX and GR play key roles in the ascorbate-glutathione cycle by reducing H_2_O_2_ to water at the expense of oxidizing ascorbate to monodehydroascorbate (MDHA) [14].

The observed increase in proline levels, a known osmoprotectant in plant cells, also supports the enhanced antioxidant responses. Also in this case, we have to underline the fact that the HDR treatments induced an enhanced antioxidant response. The occurrence of oxidative stress and activation of antioxidant mechanisms were separately reported in response to *γ*-rays and salinity stress in several other studies [11, 12, 45–47]. The extent of these responses is mainly correlated with the used doses. If the doses are too high and cells too much damaged, the antioxidant response is suppressed [11].

During IR exposure, DNA damage accumulates and proper repair mechanisms are required to maintain genome integrity [11, 47]. The SCGE analysis revealed accumulation of DNA damage in rice seeds upon exposure to *γ*-rays and salinity stress. Besides, differences were observed between the LDR and HDR treatments. Only a slight increase in DNA damage levels was evident after LDR treatment, while higher accumulation occurred in response to HDR. When the 20-day-old rice plantlets grown from seeds irradiated with HDR were analyzed, a decrease in the DNA damage levels was observed as compared with the 5-day-old seedlings and this finding suggests for the activation of DNA repair pathways. However, when the LDR treatment was considered, DNA damage repair was not observed. Similar results were recently reported in rice and *Petunia hybrida* [38, 47].

DNA repair after IR is not well understood in plants, as compared with the knowledge acquired in humans. The mechanisms are highly complex and involve multiple loci, as exemplified in *Arabidopsis* where up to 163 different genes were induced in response to 100 Gy treatment, among which 17% were related to DNA metabolism, chromosomal structure, and cell-cycle checkpoints and 11% were found to encode transcription factors [48]. Genes induced in response to IR included ATM (Ataxia Telangiectasia Mutated), involved in short-term responses to irradiation, PARP-1 and PARP-2 (poly(ADP-Ribose) Polymerase) involved in base excision repair, and the cell cycle-regulated genes RAD51, RAD54, ERCC1 and BRCA-1 [48]. The most studied DNA repair mechanism in relation to IR exposure is nonhomologous end joining (NHEJ) pathway which acts to rejoin two DNA end breaks, and involves the activity of Ku70 and Ku80 proteins. It was demonstrated that *Arabidopsis* mutants deficient in Ku80 and LIG4 (DNA ligase IV) were sensitive to *γ*-radiation and presented a delay in seed germination [49].

The present study contributes to the current discussion on the role played by the different DNA repair pathways in the plant response to IR since original data are shown concerning some key genes of the TC-NER pathway. The latter is considered a critical survival pathway, with anti-mutagenic properties, which protects cells against acute, toxic, and long-term effects of genotoxic exposure [15]. The DNA helicases XPB and XPD hereby investigated facilitate the partial unwinding of the DNA duplex leading to the recruitment of XPA, RPA, and XPG proteins and the formation of a stable preincision complex around the damage site, thus allowing the TC-NER-mediated removal of DNA damage. Orthologs of XPB and XPD helicases have been isolated in the model plant *A. thaliana *genome which contains two *XPB *genes, named *XPB1 *and *XPB2*, arranged as tandem repeats [16, 17]. The *AtXPB1 *and *AtXPB2 *genes are light-responsive and modulated in relation to development or plant tissue [17]. The *Arabidopsis xpb1 *mutant plants exhibited growth delay, lower seed viability, and loss of germination synchrony, indicating the functional redundancy of both XPB1 and XPB2 in DNA repair and transcription [17]. The XPD helicase is also shown to function in plant DNA repair, being essential for plant development and UV resistance. Liu et al. [20] found that the *Arabidopsis xpd *mutant lines were characterized by growth defects, decreased UV resistance, and limited efficiency in excision of UV photoproducts. However, no information regarding the XPB and XPD gene expression under *γ*-irradiation conditions is presently available in plants. When gene expression patterns were evaluated under IR exposure, it was shown that both *OsXPB2* and *OsXPD *were down-regulated in 5-day-old seedlings and up-regulated in 20-day-old plantlets. The observed gene expression profiles were consistent with the activation of DNA repair mechanisms in the timeframe between 5 and 20 days. On the other hand, the concomitant exposure to *γ*-rays and salinity stress resulted in gene up-regulation mainly in response to HDR treatments in both rice seedlings and plantlets. Similar expression patterns were also observed for *OsTFIIS* and *OsTFIIS-like* genes. The *MtTFIIS* gene, as well as the *MtTFIIS-like* gene, encoding a protein that shares some common features with the canonical TFIIS, was identified and characterized in *Medicago truncatula*, and its involvement in the plant response to oxidative stress was proved [22]. Recently, the *MtTFIIS* gene was also shown to be down-regulated in proliferating cell suspension treated with LDR [23]. In barrel medic cell suspension it is also shown that exposure to increased doses of *γ*-rays delivered at LDR induced accumulation of ROS/RNS with concomitant activation of antioxidant mechanisms and DNA repair pathways [23].

## 5. Conclusions

The present investigation adds valuable information concerning the complex mechanisms that regulate the plant response in case of simultaneous exposure to different abiotic stresses, evidencing at the same time the beneficial effects of IR treatments and their limited temporal efficacy in providing tolerance to adverse environmental conditions. Enhanced germination efficiency associated with increase in radicle and hypocotyl length was evident only at the early stages of plant development (5-day-old seedlings) with both LDR and HDR doses. Considering this, the potential use of *γ*-rays in seed priming still needs to be further investigated to determine the proper doses that could extend the temporal efficacy. On the other hand, the synergistic exposure to *γ*-rays and NaCl, resulted in enhanced oxidative stress and proper activation of antioxidant mechanisms, thus being compatible with plant survival. The reported data also revealed differential modulation of *OsXPB2*, *OsXPD, OsTFIIS*, and *OsTFIIS-like* genes in response to **γ**-rays and salinity stress. DNA damage accumulation and the concomitant activation of TC-NER pathway in response to these treatments were demonstrated.

## Supplementary Material

Supplementary Table 1. Oligonucleotide sequences used for qRT-PCR analysis.
Supplementary Table 2. Measurement of radicle and hypocotile length in rice 5-days-old seedlings and 20-days-old rice plantlets grown from seeds subjected to LDR and HDR *γ*-rays in absence/presence of 100 mM NaCl.
Supplementary Table 3. Genomic analysis of OsXPB2, OsXPD, OsTFIIS and OsTFIIS-like genes.
Supplementary Table 4. In silico analysis for putative protein-protein interaction.
Supplementary Figure 1. OsXPB2, OsXPD, OsTFIIS and OsTFIIS-like proteins domain organization.
Supplementary Figure 2. OsTFIIS and OsTFIIS-like protein alignments.
