# Supplementary material for: Synergistic Exposure of Rice Seeds to Different Doses of γ-Ray and Salinity Stress Resulted in Increased Antioxidant Enzyme Activities and Gene-Specific Modulation of TC-NER Pathway
Source: Biomed Res Int. 2014 Jan 16;2014:676934. doi: 10.1155/2014/676934 (PMC3914328; doi:10.1155/2014/676934)
Supplement: Supplementary file 1 — Supplementary Table 1. Oligonucleotide sequences used for qRT-PCR analysis. Supplementary Table 2. Measurement of radicle and hypocotile length in rice 5-days-old seedlings and 20-days-old rice plantlets grown from seeds subjected to LDR and HDR γ-rays in absence/presence of 100 mM NaCl. Supplementary Table 3. Genomic analysis of OsXPB2, OsXPD, OsTFIIS and OsTFIIS-like genes. Supplementary Table 4. In silico analysis for putative protein-protein interaction. Supplementary Figure 1. OsXPB2, OsXPD, OsTFIIS and OsTFIIS-like proteins domain organization. Supplementary Figure 2. OsTFIIS and OsTFIIS-like protein alignments. [file 676934.f1.doc]

**Supplementary Table 1**. Oligonucleotide sequences used for qRT-PCR analysis. Sequences are presented in 5’-3’ direction. The accession numbers are related to sequences from Rice Genome Annotation Project (<http://rice.plantbiology.msu.edu/>). Efficiency refers to the primer pair effectiveness in the QRT-PCR reaction.

| **Gene** | **Accession No.** | **Forward primer** | **Reverse primer** | **Efficiency** |
| --- | --- | --- | --- | --- |
| ***OsXPB2*** | Os01g49680 | TATGTTTGGCCACAAATGCT | TTTACCTCCAAATGCAACCA | 1.89 |
| ***OsXPD*** | Os05g0144800 | TTGCCTTTGTTGCTTCAATC | CCAATCAATGTGGCAAAGTC | 1.89 |
| ***OsTFIIS*** | Os07g12630 | GGTGATGCCTTCTCCAGAGT | ACCATCACAGCAATCCTGAA | 1.85 |
| ***OsTFIIS-like*** | Os12g06850 | AAACAGAACAACAGCATCGG | TGGCAGAATCAAGCTTCTCA | 1.81 |
| ***OsAPX*** | Os03g0285700 | GAGCCCGTGAAGGAGGAGTT | TCCCGCAAGCTGGTACAGAT | 1.82 |
| ***OsCAT*** | Os03g0163300 | ATGGACCCCTAGAAGTA | GATGAAGAAGACGGGGA | 1.88 |
| ***OsGR*** | Os03g03910 | TGTGTGCTTCGTGGATGTGTT | CCAGTCATGCTTCGGATCAGT | 1.86 |
| ***α-tubulin*** | Os11g14220 | GGTGGAGGTGATGATGCTTT | ACCACGGGCAAAGTTGTTAG | 1.87 |

**Supplementary Table 2. Measurement of radicle and hypocotile length in rice 5-days-old seedlings, as well as root and aerial part length in 20-days-old rice plantlets grown from seeds subjected to LDR and HDR *γ*-rays in absence/presence of 100 mM NaCl. Seedlings were kept on Petri dishes supplied with filter paper for germination, while plants were grown in pots containing a mixture of vermiculite, sand, and peat moss in 1:1:1 ratio and kept under greenhouse conditions (30/20^0^C day/night temperature and 12 h photoperiod with 75-80% relative humidity). Measurements were performed for 100 seedlings/plants for each experimental conditions. Results are shown in cm ± SD. Statistical differences between untreated control (NT) and irradiation doses are represented with asterisk (*p* ≤ 0.05, *).**

| **Length measurements (cm)** | | | | | | | | |
| --- | --- | --- | --- | --- | --- | --- | --- | --- |
| Plant material / Treatment | 5-days-old | | | | 20-days -old | | | |
|  | H_2_O | | NaCl | | H_2_O | | NaCl | |
|  | Radicle | Apex | Radicle | Apex | Root | Aerial part | Root | Aerial part |
| NT | 1.66±0.1 | 0.38±0.09 | 0.5±0.09 | 0.19±0.03 | 9.2±1.2 | 22.2±2.0 | 5.6±0.87 | 10.2±3.0 |
| 25 LDR | 1.86±0.05 | 0.61±0.06* | 0.54±0.06 | 0.24±0.02 | 8.7±1.0 | 24.9±2.5 | 5.8±0.98 | 7.7±3.3 |
| 50 LDR | 1.95±0.03 | 0.65±0.04* | 0.61±0.04 | 0.29±0.01 | 10.1±1.1 | 19.1±1.7 | 5.5±0.96 | 7.5±2.7 |
| 100 HDR | 2.17±0.13* | 0.72±0.03* | 0.91±0.03* | 0.32±0.01* | 11.5±0.98 | 18.2±3.0 | 4.1±0.79 | 6.9±2.9 |
| 200 HDR | 2.37±0.07* | 0.89±0.07* | 0.98±0.07* | 0.45±0.04* | 12.5±1.7 | 17.5±3.4 | 6.2±1.02 | 10.6±2.4 |

**Supplementary Table 3.** Genomic data of *OsXPB2*, *OsXPD*, *OsTFIIS* and *OsTFIIS-like* genes as indicated in the Rice Genome Annotation Project database (<http://rice.plantbiology.msu.edu/>). The intron-exon number was calculated by aligning the genomic and open reading frame (ORF) sequences on the ClustalW Program (<http://www.genome.jp/tools/clustalw/>).

| **Gene** | **Locus** | **Genomic sequence (nt)** | **Intron No** | **Exon No** | **ORF (nt)** | **Protein (aa)** |
| --- | --- | --- | --- | --- | --- | --- |
| ***OsXPB2*** | LOC_Os01g49680 | 8244 | 20 | 19 | 2301 | 766 |
| ***OsXPD*** | LOC_Os05g05260 | 5323 | 12 | 11 | 2277 | 758 |
| ***OsTFIIS*** | LOC_Os07g12630 | 3651 | 3 | 2 | 1140 | 379 |
| ***OsTFIIS-like*** | LOC_Os12g06850 | 3368 | 10 | 9 | 1023 | 341 |

This specific search resulted in the identification of one *OsXPB2* (LOC_Os01g49680) gene localized on chromosome 1, *OsXPD* (LOC_Os05g05260) gene localized on chromosome 5, and the *OsTFIIS* (LOC_Os07g12630) and *OsTFIIS-like* (LOC_Os12g06850) genes confined on chromosomes 7 and 12, respectively. The *OsXPB2* gene contains an open reading frame (ORF) of 2301nt, encoding a protein of 766aa, while *OsXPD* possesses an ORF of 2277nt which encodes for a protein of 758 aa. As for the *OsTFIIS* and *OsTFIIS-like* genes, their ORFs are of 1140 and 1023 nt, encoding for proteins of 379 and 341 aa, respectively.

**Supplementary Table 4.** *In silico* analysis for putative protein-protein interaction as shown by STRING computer program (<http://string-db.org/>).

| **Protein** | **Accession No** | **Putative protein interactions** |
| --- | --- | --- |
| **OsXPB2** | [4337788](http://string-db.org/newstring_cgi/display_single_node.pl?taskId=DqRXoTcYJ8XA&node=1472028&targetmode=proteins) | DNA repair helicase XPD |
|  | [OsI_17944](http://string-db.org/newstring_cgi/display_single_node.pl?taskId=DqRXoTcYJ8XA&node=1471472&targetmode=proteins) | TFIIH basal transcription factor complex p52 subunit |
|  | [OsJ_15417](http://string-db.org/newstring_cgi/display_single_node.pl?taskId=DqRXoTcYJ8XA&node=1469988&targetmode=proteins) | Suppressor of stem-loop protein 1 |
|  | [4328152](http://string-db.org/newstring_cgi/display_single_node.pl?taskId=DqRXoTcYJ8XA&node=1455074&targetmode=proteins) | RNA polymerase II transcription factor B subunit 4 |
|  | [4339794](http://string-db.org/newstring_cgi/display_single_node.pl?taskId=DqRXoTcYJ8XA&node=1476242&targetmode=proteins) | Cleavage and polyadenylation specificity factor |
|  | [4349131](http://string-db.org/newstring_cgi/display_single_node.pl?taskId=DqRXoTcYJ8XA&node=1496753&targetmode=proteins) | DNA excision repair protein ERCC-1 |
|  | [4331279](http://string-db.org/newstring_cgi/display_single_node.pl?taskId=DqRXoTcYJ8XA&node=1460405&targetmode=proteins) | Endonuclease |
|  | [4338726](http://string-db.org/newstring_cgi/display_single_node.pl?taskId=DqRXoTcYJ8XA&node=1474554&targetmode=proteins) | DNA-directed RNA polymerase II subunit RPB7 |
|  | [4342517](http://string-db.org/newstring_cgi/display_single_node.pl?taskId=DqRXoTcYJ8XA&node=1481815&targetmode=proteins) | RNA polymerase subunit |
| **OsXPD** | [OsJ_03086](http://string-db.org/newstring_cgi/display_single_node.pl?taskId=IpFzONlnBjij&node=1452463&targetmode=proteins) | DNA repair helicase XPB2 |
|  | [OsJ_15417](http://string-db.org/newstring_cgi/display_single_node.pl?taskId=IpFzONlnBjij&node=1469988&targetmode=proteins) | Suppressor of stem-loop protein 1 |
|  | [4328152](http://string-db.org/newstring_cgi/display_single_node.pl?taskId=IpFzONlnBjij&node=1455074&targetmode=proteins) | RNA polymerase II transcription factor B subunit 4 |
|  | [OsI_17944](http://string-db.org/newstring_cgi/display_single_node.pl?taskId=IpFzONlnBjij&node=1471472&targetmode=proteins) | TFIIH basal transcription factor complex p52 subunit |
|  | [4334040](http://string-db.org/newstring_cgi/display_single_node.pl?taskId=IpFzONlnBjij&node=1465017&targetmode=proteins) | Putative cyclin |
|  | [4339794](http://string-db.org/newstring_cgi/display_single_node.pl?taskId=IpFzONlnBjij&node=1476242&targetmode=proteins) | Cleavage and polyadenylation specificity factor |
|  | [4349131](http://string-db.org/newstring_cgi/display_single_node.pl?taskId=IpFzONlnBjij&node=1496753&targetmode=proteins) | DNA excision repair protein ERCC-1 |
|  | [4331279](http://string-db.org/newstring_cgi/display_single_node.pl?taskId=IpFzONlnBjij&node=1460405&targetmode=proteins) | Endonuclease |
|  | [4338271](http://string-db.org/newstring_cgi/display_single_node.pl?taskId=IpFzONlnBjij&node=1473321&targetmode=proteins) | MSH-like DNA mismatch repair protein |
| **OsTFIIS** | KOG2691 | RNA polymerase II subunit 9 |
|  | KOG0214 | RNA polymerase II, second largest subunit |
|  | KOG0260 | RNA polymerase II, large subunit |
|  | KOG4392 | RNA polymerase II, L subunit |
|  | KOG1522 | RNA polymerase II, subunit POLR2C/RPB3 |
|  | KOG3281 | RNA polymerase, 25-kDa subunit |
|  | KOG3490 | Transcription elongation factor SPT4 |
|  | KOG1999 | RNA polymerase II transcription elongation factor DSIF/SUPT5H/STP5 |
|  | KOG2905 | Transcription initiation factor IIF, small subunit (RAP30) |

The *in silico* analysis for putative protein-protein interaction revealed that the OsXPB2 and OsXPD proteins interact with each other and mainly with same type or proteins like different subunits of TFIIH (p52 and the suppressor of stem-loop protein 1) and RNA polymerase II (RPB7,the RNA polymerase II transcription factor B subunit 4) as well as with other proteins involved in different DNA repair pathways (DNA excision repair protein ERCC-1 and MSH-like DNA mismatch repair protein) [1, 2]. As for the OsTFIIS protein, the STRING analysis showed that it mostly interacts with several subunits of RNA polymerase II (e.g. 9, L, POLR2C/RPB3, 25-kDa subunit) and other transcription factors (SPT4, DSIF/SUPT5H/STP5, TFIIF subunit RAP30). In the case of OsTFIIS-like protein, no information was able to be retrieved through this program, since the sequence is not characterized in neither prokaryotic nor eukaryotic organisms. Further analyses on protein expression, characterization and experimental protein-protein interactions are still in progress.

**Supplementary Figure 1.** OsXPB2, OsXPD, OsTFIIS and OsTFIIS-like proteins domain organization as evidenced by using the CDD (Conserved Domain Database) program (<http://www.ncbi.nlm.nih.gov/cdd/>).


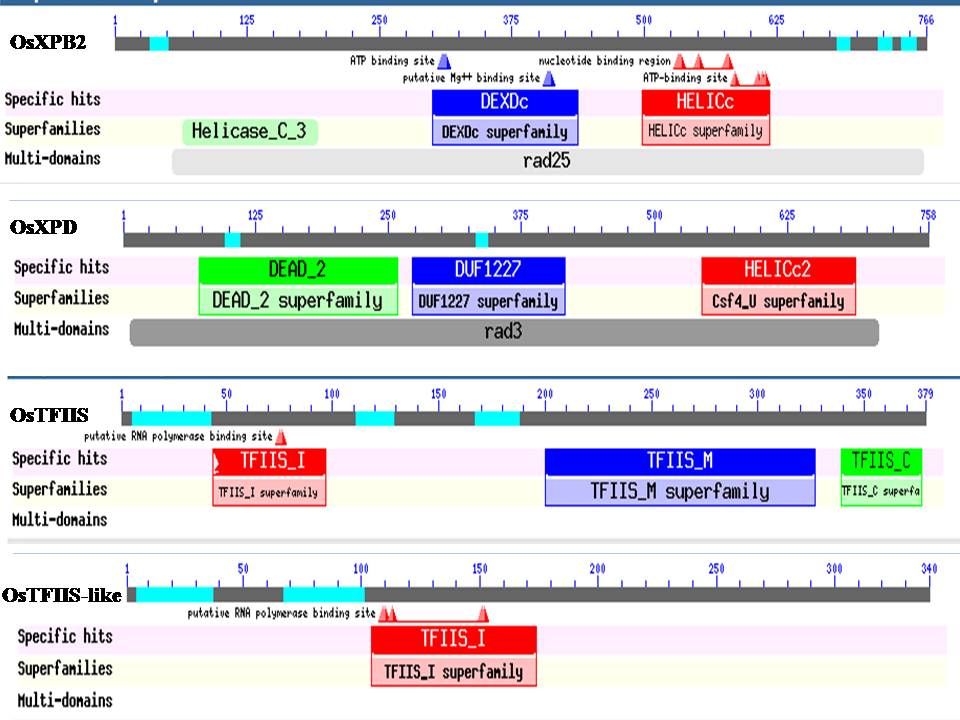


The protein conserved domain search was performed by using the CDD (Conserved Domain Database) program (<http://www.ncbi.nlm.nih.gov/cdd/>). OsXPB2 and OsXPD proteins contain specific domains usually found in most helicases, like DEAD or DEXD domains and helicase C-terminal domain [3]. Additionally, the protein contains also ATP binding sites and nucleotide binding regions. The OsTFIIS protein comprises the three specific domains encountered in all eukaryotic organisms, namely the N-terminal domain I, involved in nuclear targeting, the central domain II, playing a role in the interaction between TFIIS and RNA polymerase II, and the C-terminal domain III, which is essential for the stimulation of RNA cleavage [4]. On the other hand, the OsTFIIS-like protein possesses only the N-terminal domain I. characteristic to several other transcription elongation factors such as Elongin A and CRSP70 complex [5]. In addition, the two protein sequences also contain putative RNA polymerase binding sites.

**Supplementary Figure 2. (A)** OsTFIIS and OsTFIIS-like protein alignments performed by using the ClustalW Program (<http://www.genome.jp/tools/clustalw/>). Red box, N-terminal domain I; blue box, central domain II; green box, C-terminal domain III. **(B)** Alignment between OsTFIIS and OsTFIIS-like N-terminal domain I. In red are represented the conserved lysine (L) and tryptophan (W) residues. Conserved amino acids are marked by asterisks ("*") when the residues are identical in all sequences in the alignment, by two points (":") for conserved substitutions and one point (".") for semi-conserved substitutions.

**(A)**

**
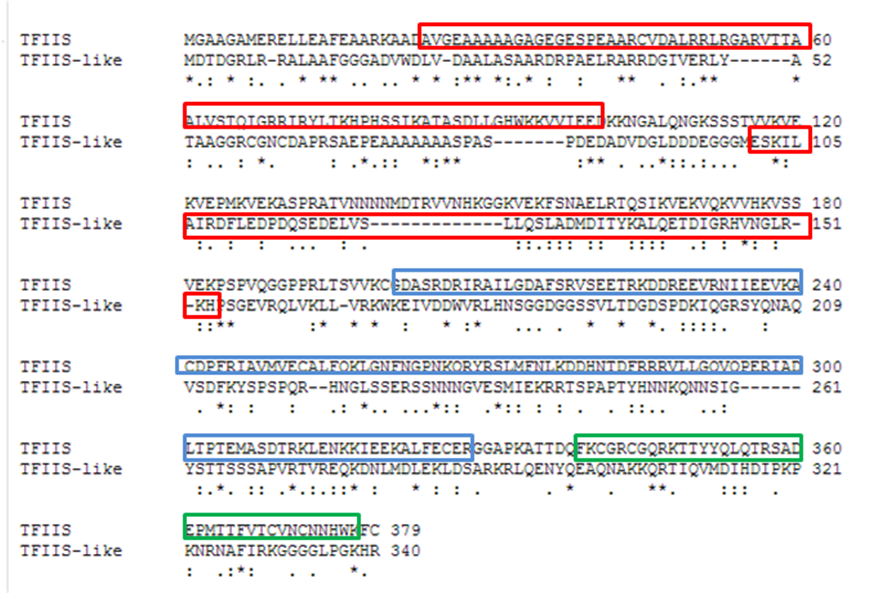
**

**(B)**


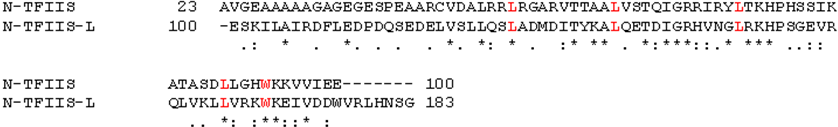


When the OsTFIIS and OsTFIIS-like sequences were aligned, only a 14% identity between the two proteins was observed. However, the percentage of similarity increased inside the N-terminal domain, reaching to 25%. This domain is characterized by the presence of the LW motif, specifically conserved among transcription elongation factors, containing four lysine residues and one tryptophan residue [5].

The existence of different TFIIS proteins was reported in vertebrate species. The mouse and human SII genes (TCEA1, TCEA2 and TCEA3) show distinct expression patterns across tissues [6]. Uzureau et al. [7] identified two trypanosome (*Trypanosoma brucei*) proteins (TbTFIIS1, TbTFIIS2-1) with homology to TFIIS. These proteins show different structural features, since TbTFIIS1 contains only the canonical domains II and III while TbTFIIS2-1 contains also the domain I and a PWWP (Proline-Tryptophan-Tryptophan-Proline) domain, required for the binding of DNA methyltransferases to heterochromatin. As for plants, the ET1 protein, found in maize chloroplasts [8], and the FACT (Facilitates Chromatin Transcription) complex in *Arabidopsis* [9] were shown to possess a high sequence similarity to the eukaryotic TFIIS. The *MtTFIIS* gene, as well as the *MtTFIIS-like* gene, encoding a protein that shares some common features with the canonical TFIIS, were identified and characterized in *Medicago truncatula*, and their involvement in the plant response to oxidative stress was proved [5].

**References**

1. Xu, H., et al., *Plant homologue of human excicion repair gene ERCC1 points to conservation of DNA repair mechanisms*, Plant J, 1998. **13**(6):p. 823-829.

2. Culligan, K.M. and J.B. Hays, *DNA mismatch repair in plants (An Arabidopsis thaliana gene that predicts a protein belonging to the MSH2 subfamily of eukaryotic MutS homologs)*, Plant Phyiol, 1997. **115**(2):p. 2833-2839.

3. Umate, P., et al., *Architectures of the unique domains associated with the DEAD-box helicase motif*, Cell Cycle, 2010. **15**(20):p. 4228-4235.

4. Awrey, D.E., et al., *Yeast transcription elongation factor (TFIIS), structure and function*, J Biol Chem, 1998. **273**:p. 22595-22605.

5. **Macovei, A., et al.,** *The TFIIS and* TFIIS-like *genes from* Medicago truncatula *are involved in oxidative stress response*, Gene, 2011. **470**:p. 20-30.

6. Labhart, P. and G.T. Morgan, *Identification of novel genes encoding transcript elongation factor TFIIS (TCEA) in vertebrates: conservation of three distinct TFIIS isoforms in frog, mouse and human*, Genomics, 1998. **52**:p. 278-288.

7. Uzureau, P., et al., *Identification and characterization of two trypanosome TFIIS proteins exhibiting particular domain architectures and different nuclear localization*, Mol Microbiol, 2008. **69**:p. 1121-1136.

8. da Costa-Nunes, J.A., et al., *Characterization of the three Arabidopsis thaliana RAD21 cohesins revealed differential responses to ionizing radiation*, J Exp Bot, 2006. **57**:p. 971-983.

9. Belotserkovskaya, R., et al., *FACT facilitates transcription – dependent nucleosome alteration*, Science, 2003. **310**:p. 1090-1093.
